# Decentralizing oxygen availability and use at primary care level for children under-five with severe pneumonia, at 12 Health Centers in Ethiopia: a pre-post non-experimental study

**DOI:** 10.1186/s12913-022-08003-4

**Published:** 2022-05-19

**Authors:** Habtamu Seyoum Tolla, Dinkineh Bikila Woyessa, Rahel Belete Balkew, Yigeremu Abebe Asemere, Zinabie Feleke Fekadu, Alemayehu Berhanu Belete, Martha Gartley, Audrey Battu, Felix Lam, Alebel Yaregal Desale

**Affiliations:** 1 Clinton Health Access Initiative, Addis Ababa, Ethiopia; 2 Clinton Health Access Initiative, Boston, USA

## Abstract

**Background:**

Pneumonia is the leading infectious cause of death in children worldwide, accounting for 15% of all deaths in children under the age of five. Hypoxemia is a major cause of death in patients suffering from pneumonia. There is strong evidence that using pulse oximetry and having reliable oxygen sources in health care facilities can reduce deaths due to pneumonia by one-third. Despite its importance, hypoxemia is frequently overlooked in resource-constrained settings. Aside from the limited availability of pulse oximetry, evidence showed that healthcare workers did not use it as frequently to generate evidence-based decisions on the need for oxygen therapy. As a result, the goal of this study was to assess the availability of medical oxygen devices, operating manuals, guidelines, healthcare workers’ knowledge, and skills in the practice of hypoxemia diagnosis and oxygen therapy in piloted health centers of Ethiopia.

**Methods:**

A pre-post non-experimental study design was employed. An interviewer-administered questionnaire was used to collect primary data and review medical record charts. A chi-square test with a statistical significance level of *P* < 0.05 was used as a cut-off point for claiming statistical significance.

**Results:**

Eighty one percent of healthcare workers received oxygen therapy training, up from 6% at baseline. As a result of the interventions, knowledge of pulse oximetry use and oxygen therapy provision, skills such as oxygen saturation and practices of oxygen therapy have significantly improved among healthcare workers in the piloted Health Centers. In terms of availability of oxygen devices (e.g. cylinders, concentrators, and pulse oximeters) in the facilities, seven (58%) facilities did not have any at baseline, but due to the interventions, all facilities were equipped with the oxygen devices.

**Conclusions:**

Given the prevalence of pneumonia and hypoxemia, a lack of access to oxygen delivery devices, as well as a lack of knowledge and skills among healthcare workers in the administration of oxygen therapy, may represent an important and reversible barrier to improving child survival. Therefore, scaling up clinician training, technical support, availability of oxygen devices, guidelines, manuals, strengthening maintenance schemes, and close monitoring of healthcare workers and health facilities is strongly advised.

**Supplementary information:**

The online version contains supplementary material available at 10.1186/s12913-022-08003-4.

## Introduction

Pneumonia is the single most common infectious cause of death in children, accounting for 15% of all deaths in children under the age of five [1]. Hypoxemia, or a low level of oxygen in the blood, is a major fatal complication of pneumonia, and the risk of death increases as the severity of hypoxemia increases [2–5]. According to a systematic review, 13.3% of children with pneumonia have hypoxemia [6]. Furthermore, according to the UN IGME report, 23% of the 5.9 million annual child deaths are caused by neonatal conditions such as birth asphyxia, sepsis, and low birth weight, all of which can present with hypoxemia [4]. Clinical manifestations of hypoxemia are insensitive [3, 7]. Many children with hypoxemia are missed if pulse oximetry is not used on a regular basis [3, 8].

Countries are gaining experience in the clinical, biomedical, and training aspects of establishing and maintaining effective oxygen delivery systems in hospitals and lower-level health facilities [4, 9]. There is evidence that the use of pulse oximetry and the availability of reliable oxygen sources in district hospitals can reduce pneumonia death rates by about one-third [4, 9]. Similarly, improving access to oxygen devices (oxygen concentrator and cylinder) and pulse oximetry has been shown to reduce childhood pneumonia mortality by up to 35% in high-burden child pneumonia settings [5, 8].

Despite its importance in almost all types of severe illness, hypoxemia is frequently under-recognized and under-managed in resource-constrained settings. For a significant number of critically ill children admitted to hospitals in low-resource settings, oxygen therapy remains an unattainable luxury [4]. An assessment report in Ethiopia revealed that functional oxygen service availability and pulse oximetry use are very low, with only 2% of Health Centers (HCs) having a fully functional cylinder and/or concentrator available, and no functional pulse oximeter available in 314 (100%) of the HCs visited for the assessment [10]. This is especially true for patients in lower-level health facilities, such as HCs, where supplies are frequently unreliable and treatment benefits may be unavailable or diminished due to poorly maintained, inappropriate equipment, poorly trained staff, or inadequate guidelines [4].

Raising the knowledge and skills on these topics is likely to have a significant impact on clinical and public health practices. Health professionals should be cognizant of the clinical signs and symptoms that indicate the presence of hypoxemia. More widespread use of pulse oximetry, as a noninvasive measure of arterial oxygen saturation, could lead to more reliable detection of hypoxemia. Oxygen therapy must be widely available; even in remote areas, this can be accomplished through the use of oxygen concentrators, which can run on either regular or alternative power sources [4].

However, evidence showed that, in addition to the limited availability of pulse oximetry in health facilities, Health Care Workers (HCWs) did not use it as frequently for children with severe pneumonia and thought it was less important for diagnosis than other tools in developing-country clinical settings [3, 11]. Furthermore, to the best of our knowledge, there is no published evidence about its availability and practice at health centers in Ethiopia, despite the fact that HCs are a critical component of the primary health care unit, with each HC serving an estimated total and under-five population of 25,000 and 4,000, respectively. It also offers critical child health services such as Integrated Management of Newborn and Child Health Illnesses (IMNCI). According to unpublished reports, oxygen concentrators and pulse oximetry are only available and used at the hospital level. As a result, the goal of this study is to assess the availability of medical oxygen devices, investigate the knowledge and skills of HCWs in diagnosing hypoxemia and provide medical oxygen therapy in 12 pilot HCs in Ethiopia.

## Methods

To assess the availability of medical oxygen devices and assess the knowledge, skills and practices of HCWs in the application and use of oxygen therapy, baseline assessment was conducted in 12 HCs in four agrarian regions of Ethiopia. Based on the assessment, baseline values were set and interventions such as procurment of oxygen concentrators, pulse oximeters, development of oxygen therapy manuals and flowchart algorithms for children and adults and oxygen therapy trainings and supportive supervision were conducted.

### Study design, period, setting and population

The study employed a pre-post survey design. The study was carried out in 12 HCs located in four agrarian regions of Ethiopia, namely Amhara, Oromia, SNNP, and Tigray, from February to September 2019. The baseline data were collected in February 2019, followed by consecutive months of technical support and mentorship from May to September 2019. The study setting consisted of 12 high-volume HCs that were purposefully chosen to meet the geographical equity of HCs in their respective regions. Additionally, HCWs working in the piloted HCs were the study populations. Furthermore, medical record reviews of children aged 0 to 59 months with severe pneumonia cases were reviewed to monitor clinical practices of HCWs, including routine use of pulse oximetry and administration of oxygen.

### Sample size and sampling procedures

All 12 pilot HCs were included in the study because the number of pilot HCs were insufficient to apply a sampling procedure. The previous four months of medical records were reviewed retrospectively beginning from the date of the visit and included all pneumonia cases within that timeframe to see how the cases were managed with respect to oxygen administration prior to the provision of any device, training, and mentorship. Following the baseline assessment, the pilot facilities received the necessary training on hypoxemia diagnosis and oxygen therapy in children based on a WHO-adopted national training manual. All HCs were given pulse oximeter and oxygen concentrators. On-site mentoring was also offered to improve HCWs knowledge and skills. Medical records of all severe pneumonia cases were retrospectively reviewed, in three-month intervals. A total of 2,960 medical records of children were reviewed. Furthermore, 36 HCWs’ knowledge and skills were assessed prospectively.

### Data collection methods and tools

Structured surveys were used to collect primary data from HCWs and extract secondary data from medical record reviews. The methods listed below were used to collect data. To collect the variables of interest, structured data extraction tools were created. SurveyCTO (Dobility Inc, Cambridge, MA USA), an electronic data collection software, was programmed with the data collection tools, and the software application was downloaded onto tablet computers.

### Medical record review

The review of medical records were conducted using standard, structured, and closed-ended questionnaires based on WHO guidelines [4]. Every three months, chart reviews were performed to assess the status of medical oxygen therapy for pediatric patients. Baseline values were set by reviewing medical records retrospectively. Following the implementation of the interventions, medical records were reviewed twice. Because the volume of cases was small, all severe pneumonia cases identified in that specific HC during the review period were included. Following that, the procedures and applications of oxygen therapy were evaluated, including oxygen check-ups, saturation records, oxygen prescriptions if hypoxemic, flow rates, routes of administration, follow-up status, and the child’s outcomes. Technical support and feedback were provided to HCWs based on the gaps identified in the chart reviews. The reviewers of the medical records were health care professionals who have a minimum of a second degree in health science and have a wealth of experience in the field.

### Interview

To assess HCW knowledge and skills, the interview guide used both closed and semi-structured open-ended questionnaires. The interview guide includes questions about their knowledge, skills, and applications of pulse oximetry and oxygen concentrators. Respondents for the observations were purposefully chosen based on their specific roles. Eligible respondents were initially identified in health facilities and contacted, with the purpose of the study explained. At the baseline assessment, the interviewees were health professionals assigned to oxygen treatment units.

### Observations

The observation method was also used to assess HCWs’ skill and practice on volunteers because it can be empirically calibrated by observations on normal volunteers [12]. This reinforces and aids in the triangulation of data gathered through chart reviews and demonstration of skill and practices of HCWs.

### Interventions

Based on the baseline assessment, oxygen devices such as oxygen concentrators and pulse oximetry were procured and provided. Moreover, oxygen therapy manuals for children and adults, oxygen flow-chart algorithms, oxygen therapy trainings for clinicians, supportive supervisions and technical support were provided. Similarly, to ensure the availability of medical oxygen, a device maintenance system was designed in the context of primary health care units which enables them to easily link with biomedical engineers and technicians available either at woreda health office, zone health department, regional health bureau, or nearby hospitals.

### Data analysis

MS-Excel Office 2016 was used to clean the data. Statistical Packages for Social Sciences were also used for additional cleaning and preparation (SPSS). The primary outcomes of the study were as follows: (1) the proportion of children under the age of five admitted with pneumonia who had a blood oxygen saturation measurement (SpO2); (2) the proportion of children under the age of five admitted with severe pneumonia and with SpO2 < 93% that received oxygen; (3) the proportion of facilities with an oxygen concentrator and a pulse oximeter; and (4) the knowledge and skills of HCWs on oxygen therapy practice. To present key findings, a descriptive analysis was used to generate frequencies, tables, and charts. Chi-squared tests were used to determine whether there were statistical differences between consecutive follow-up periods. A p-value of less than 0.05 was used as a cut-off point to determine whether or not there was a relationship.

### Ethical clearance

 The research protocol was reviewed, and ethical approval was obtained from the Ethiopian Public Health Institute (EPHI) under protocol number EPHI-IRB-175-2019, with a letter of approval dated 19 June 2019 and referencing EPHI13.6/136. A confirmation/clearance letter was also obtained from the Ministry of Health (MoH) and Regional Health Bureaus (RHBs). All participants in the study gave their verbal consent. There was no known risk in the study, and no payment was given to participants.

## Results

### Health facilities background information

**Fig. 1:**
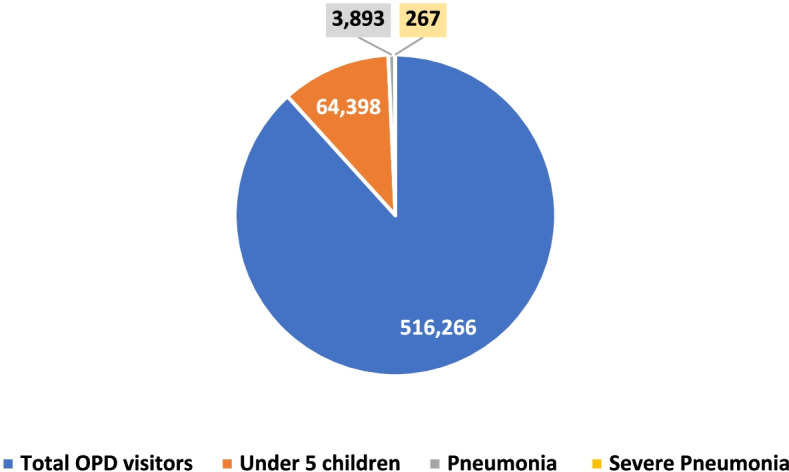
Disaggregation of outpatient visitors and cases. Data on outpatient visitors and pneumonia cases

The pilot HCs are located in four agrarian regions. The distance between the HCs and the nearest referral hospital ranges from 1 to 60 km. During the study period, 516,266 people visited the outpatient department (OPD). Out of the total OPD visitors (Fig. 1), 64,398 (12.5%) were children under the age of five; among these under-five visitors, 3,893 (6%) and 267 (0.41%) had pneumonia and severe pneumonia cases, respectively. The average number of health professionals assigned to work in the outpatient departments of the pilot HCs were 2.25 per HC.

In terms of the availability of oxygen devices (cylinders, concentrators, and pulse oximeters) in the facilities, seven (58%) facilities did not have any at baseline, but due to the interventions, all facilities were equipped with the oxygen devices. A total of 2,960 medical records were reviewed. Of the total medical records, 58% and 42% were male and female, respectively. In general, 240 (8%), 1203 (41%), and 1513 (51%) of the total number of medical records reviewed fall within the age categories of < = 1month, > 1-11months, 11–59 months and 4 patients chart had missing data on age (Table 1).

**Table 1:** Medical record review by region and health center

Name of HC	Region				Total	Baseline (Feb-2019)	May 2019	End-line (September 2019)
	Amhara	Oromia	SNNPR	Tigray				
Kombolcha HC	250				**250**	103	48	99
Woreta HC	429				**429**	257	85	87
Bahir Dar HC	265				**265**	116	73	76
Alemgena HC		116			**116**	23	44	49
Chefe Donsa HC		298			**298**	96	97	105
Sebeta HC		242			**242**	103	75	64
Millenium HC			93		**93**	38	33	22
Wondogenet HC			118		**118**	22	60	36
Yirgalem HC			234		**234**	67	121	46
Edaga Hamus HC				44	**44**	13	21	10
Megab HC				298	**298**	83	131	84
Wukro HC				573	**573**	202	191	180
**Total**	**944**	**656**	**445**	**915**	**2960**	**1123**	**979**	**858**

### Knowledge assessment

The majority (94%) of professionals in the pilot health facilities did not have oxygen therapy training at baseline, and as part of our designed interventions, 29 (81%) HCWs received oxygen therapy training in the pilot health facilities.

Based on HCWs’ knowledge assessment of whether clinical signs are reliable predictors of hypoxemia or not, our study revealed that only 14% of HCWs correctly answered at baseline. However, after training and technical assistance, the majority 30(83%) of them responded that clinical signs are not a reliable predictor of hypoxemia. Furthermore, when HCWs were asked whether hypothermia and poor peripheral perfusion would affect the saturation measurement of pulse oximetry, the majority 34(94%) correctly responded. However, only 19% of them were able to respond correctly at the start.

**Fig. 2:**
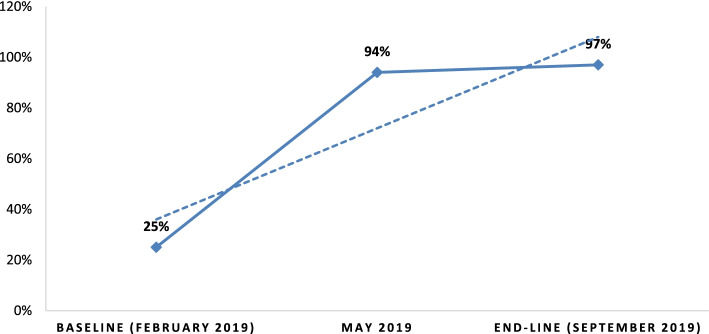
Knowledge of HCWs on the cut-off point of oxygen saturation to initiate oxygen therapy. Assessment of the knowledge of HCWs on the cut of point of oxygen saturation to initiate oxygen therapy at baseline, mid-term and end-line (the dotted line shows the trend over time)

In terms of HCWs’ knowledge of the cut-off point of oxygen saturation to initiate oxygen therapy (i.e. SPO2 <93%), 9(25%), 34(94.4%), and 35(97%) correctly stated the cut-off point at baseline (February 2019), May 2019, and end-line (September 2019), respectively. Because of ongoing capacity building, the majority of HCWs were aware of the recommended cut-off point for initiating oxygen therapy. As a result, the intervention resulted in a significant improvement over the baseline finding (Fig. 2).

**Fig. 3:**
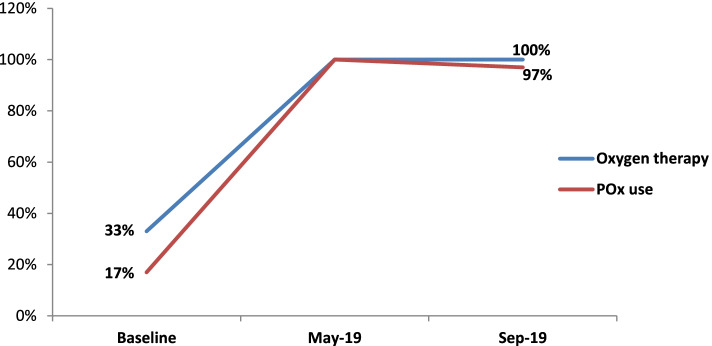
Trends in pulse oximetry use and oxygen therapy provision. Depicts the trends in pulse oximetry use and oxygen therapy at baseline, mid-term and end-line

The study revealed that after the intervention measures were implemented, oxygen therapy services increased dramatically and were available in all 12 HCs. However, only 4 (33%) of the HCs were able to provide oxygen therapy services at the outset. Similarly, the majority (97%) of HCWs across all regions used pulse oximetry to measure oxygen saturation, compared to only 17% at baseline. The provision of oxygen therapy services in HCs and the use of pulse oximetry among HCWs yielded statistically significant (*p* < 0001) results (Fig. 3).

**Fig. 4:**
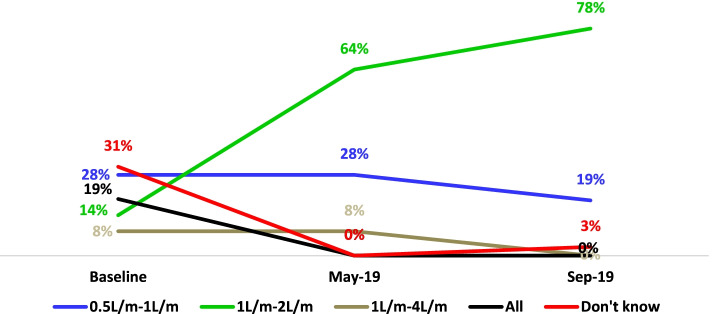
Knowledge of HCWs about the standard flow rate of oxygen administration for infants. Depicts the knowledge of HCWs on the standard flowrate of oxygen administration for infants at baseline, mid-term and end-line

The study also assessed HCWs’ knowledge of the standard flow rate of oxygen administration for infants (1-2LPM), with the majority (78%) correctly responding that the standard flow rate which was only 14% at baseline, it also yields a statistically significant result (*p* < 0001). HCWs who misunderstood the standard flow rate of oxygen administration for infants, on the other hand, sharply declined from the baseline, as shown in the line graph (Fig. 4).

### Skill assessment

**Fig. 5:**
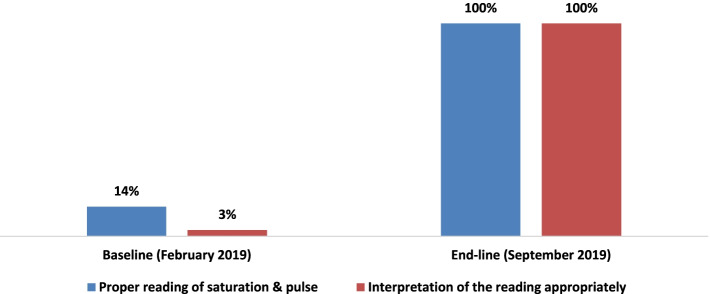
Proper reading and interpretation of the reading of saturation and pulse at baseline consecutive follow-ups. Shows the HCWs knowledge and skill on proper reading and interpretation of the reading of saturation and pulse at baseline consecutive follow-ups at baseline and end-line

According to the skill assessments of HCWs, all HCWs were able to properly place the pulse oximetry probe on the finger after training and subsequent technical support, but only 5(14%) were able to do so at baseline. Similarly, based on the skill assessment for the application of pulse oximetry, all HCWs knew to wait until the waveform became stable, but only 38% did so at baseline. Additionally, findings revealed that all HCWs were able to correctly read the oxygen saturation after mentorship was provided. However, only 5(14%) of HCWs were able to correctly read the oxygen saturation at baseline. Similarly, appropriate interpretation increased significantly from 3% at baseline to 100% in subsequent rounds of capacity building and mentorship support (Fig. 5).

 Our study also depicted that the majority of HCWs 26(72%) were unable to demonstrate nasal prong placement at baseline, but with the technical and capacity-building supports provided, all HCWs were able to properly do so. Similarly, almost all HCWs 35(97%) were unable to properly set the flow rates for nasal prongs at baseline, but this was reversed after the intervention was implemented, and all HCWs were able to properly set flow rates.

### The practice of oxygen therapy

**Fig. 6:**
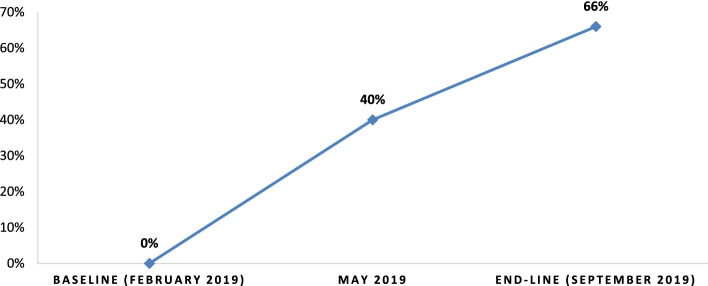
Oxygen saturation (SpO2) records at baseline, consecutive follow-ups. Depicts the oxygen saturation (SpO2) records at baseline, consecutive follow-ups at baseline, mid-term and end-line

According to our findings, HCWs’ practice of recording oxygen saturation has significantly improved. The practice of recording oxygen saturation levels, as shown in the bar graph below, shows progressive improvement following interventions. Although the practice of recording oxygen saturation was absent at the start, documentation of SPO2 for pediatric patients at triage and/or at initial assessment increased from 0% to 66% as a result of capacity building training and technical support to HCWs (Fig. 6).

The findings revealed that 420 (37%) of the total baseline records reviewed did not have a respiratory rate measured at diagnosis. The respiratory rates of 111 (11%) and 171 (20%) of the cases were not recorded after the intervention in the consecutive follow-up period. Overall, there were improvements when compared to the baseline, but there were still some room for improvement.

**Fig. 7:**
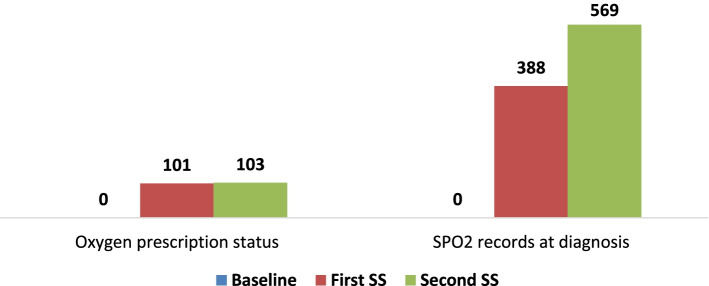
Status of oxygen prescription and SPO2 records at diagnosis (at triage and/or at initial assessment). Shows the status of oxygen prescription and SPO2 records at diagnosis (at triage and/or at initial assessment)at baseline and end-line

In terms of SPO2 measurement record for patients at triage and/or at initial assessment, SPO2 was not recorded at all at baseline, but it was recorded for 388 (40%) and 569 (66%) patients in the follow-up periods. Of the children whose oxygen saturation levels were measured, 68 and 55 were hypoxemic. The result is displayed in (Fig. 7).

## Discussion

The study’s findings revealed a lack of any of the oxygen devices in more than half of the facilities and limited capacity of HCWs for oxygen therapy across all 12 HCs. In almost all facilities, HCWs’ abilities to provide oxygen therapy services were severely limited, with significant gaps in knowledge and skill in the use of oxygen concentrators and pulse oximetry. As a result of the identified gaps, oxygen trainings for clinicians were provided, increasing the availability of trained HCWs from 6% to 81%. In addition to the availability of trained HCWs, their knowledge of the application of oxygen therapy and pulse oximetry use increased from 33% to 100% and 17% to 97%, respectively in the pilot HCs, respectively.

Studies have found that low knowledge and skills among HCWs were almost universally reported that many HCWs had misconceptions and fears about oxygen therapy, which hampered their motivation. These studies also stated that the projects providing initial training have demonstrated that HCWs’ knowledge and skills had improved [13–17]. Furthermore, researchers documented that a lack of guidelines, equipment, and technical difficulties affect HCWs motivation and that these challenges can be addressed through on-site training and regular on-site support [13, 14, 18–20]. In line with this, our study identified knowledge and skill gaps among HCWs and provided interventions such as oxygen equipment, on-site training, and regular mentorship, which significantly improved HCWs’ knowledge and skill in providing oxygen therapy services in the facilities. Furthermore, the HCWs stated in a qualitative interview that the interventions reduced unnecessary referrals to nearby district hospitals.

A study that looked at the barriers to using oxygen and pulse oximetry in children with pneumonia found that lack of training and guidelines were two of the most frequently cited barriers [3]. Similarly, our study found that the majority of HCWs lacked knowledge and skills in using oxygen devices and pulse oximetry at the outset. Furthermore, our baseline study discovered that nearly three-quarters of HCWs believe clinical signs are a reliable predictor of hypoxemia, despite numerous evidences [3, 7, 21, 22]. As a result, interventions such as training and technical support were provided to improve the quality of services and, ultimately, the health outcomes of pediatric patients. These interventions reversed HCWs’ misperceptions that clinical signs are reliable predictors of hypoxemia and significantly increased their knowledge.

### Limitation

Although the HCs were chosen from four regions of Ethiopia, they were not chosen at random; rather, they were chosen based on patient caseload. Although the study included all the pilot HCs, the findings of the study may not be generalizable at a large scale since the number of health centers are few. As a result, our findings should be applied with caution to HCs with similar settings.

## Conclusions

Due to the effects of the multi-faceted interventions, the availability of medical oxygen devices, oxygen therapy manuals for children and adults and oxygen flow-chart algorithms have significantly changed from the baseline values. Similarly, the knowledge and skill of health care workers in proper use and practice of medical oxygen therapy has significantly improved in the pilot HCs.

In general, our findings can help practitioners and policymakers understand how to improve oxygen therapy in HCs, as well as lower level and key components of primary care units that are expected to provide comprehensive child health services like IMNCI. Given the prevalence of pneumonia and hypoxemia, a lack of access to oxygen delivery devices, as well as a lack of knowledge and skill among HCWs in the administration of oxygen therapy, may represent an important and reversible barrier to improved child survival. Scaling up training, technical support, and close monitoring of HCWs and health facilities is therefore strongly advised.

## Supplementary Information

**Additional file 1.** Health Center Profile.

**Additional file 2.** HCWs.

**Additional file 3.** HCW Skill.

**Additional file 4.** Medical equipment mgt.

**Additional file 5.** Medical record review Tool.

**Additional file 6.** Checklist for Chart Review.

**Additional file 7.** HC_MRR_TOOL_Baseline.

**Additional file 8.** HCW_Skill_Asses_Baseline.

**Additional file 9.** HC_MRR_TOOL_1st Round.

**Additional file 10.** HCW_Knowledge_Asses_1st Round.

**Additional file 11.** HCW_Skill_Asses_1st Round.

**Additional file 12.** HC_MRR_TOOL_2nd Round.

**Additional file 13.** HCW_Knowledge_Asses_2nd Round.

**Additional file 14.** HCW_Skill_Asses_2nd Round.

## Data Availability

All data generated or analyzed during this study are included in this published article and its supplementary information files.
