# Supplementary material for: Decentralizing oxygen availability and use at primary care level for children under-five with severe pneumonia, at 12 Health Centers in Ethiopia: a pre-post non-experimental study
Source: BMC Health Serv Res. 2022 May 19;22:676. doi: 10.1186/s12913-022-08003-4 (PMC9121544; doi:10.1186/s12913-022-08003-4)
Supplement: Supplementary file 1 — Additional file 1. Health Center Profile. [file 12913_2022_8003_MOESM1_ESM.docx]

**Section I.1 HEALTH FACILITY PROFILE**

1. Region ______________________
2. Zone ________________________
3. Woreda _____________________
4. Name of Health Centre _________________________
5. Estimated total number of population in the catchment to be served_____________________
6. Total number of health posts in the catchment of the HC_____________________
7. Name of the nearby hospital for referral-------------------
8. Distance from the HC to the nearby hospital in KM____________
9. Total number of health professionals in the facility______________-
10. Number of professionals dedicated to work in under 5 OPD_______________
11. Number of professionals dedicated to work in EOPD_________
12. Total number of OPD visitors in the last three months (Ginbot 1 to Hamle 30, 2011) ________________
13. Total number of Under-5 children OPD visitors in the last three months (Ginbot 1 to Hamle 30, 2011) _____________________
14. Total number of EOPD visitors in the last three months (Ginbot 1 to Hamle 30, 2011) ____________________
15. Total number of Under-5 children pneumonia cases in the last three months (Ginbot 1 to Hamle 30, 2011) _____________
16. Total number of Under-5 children severe pneumonia cases in the last three months (Ginbot 1 to Hamle 30, 2011)___________________
17. Total # of Under-2 month children Birth Asphyxia cases in the last three months (Ginbot 1 to Hamle 30, 2011)___________
18. Total # of Under-2 month children Very low birth Wight/ Very preterm in the last three months (Ginbot 1 to Hamle 30, 2011)_________
19. Total # of Under-2 month children Very Severe Disease cases in the last three (Ginbot 1 to Hamle 30, 2011)­­­­­­­­­­­­­­­___________
20. Total number of emergency beds in the HC_________
