# Supplementary material for: Decentralizing oxygen availability and use at primary care level for children under-five with severe pneumonia, at 12 Health Centers in Ethiopia: a pre-post non-experimental study
Source: BMC Health Serv Res. 2022 May 19;22:676. doi: 10.1186/s12913-022-08003-4 (PMC9121544; doi:10.1186/s12913-022-08003-4)
Supplement: Supplementary file 2 — Additional file 2. HCWs. [file 12913_2022_8003_MOESM2_ESM.docx]

**Section I.2 Self-administered question for knowledge assessment**

**Instructions**

## Talk to **three healthcare providers** who are assigned and currently working in **under five OPD, labor and delivery and the head of health centre**

- Introduce yourself and discuss the purpose of the assessment
- Obtain verbal consent for participation before proceeding to asking questions

**Background Information**

1. Position _______________________
2. Sex
3. Male
4. Female
5. Profession _________________________
6. Level of education
7. Certificate
8. Diploma
9. Degree
10. Masters
11. MD
12. Other
13. Years of experience ____________________
14. Duration of work in the health center ___________
15. Duration of work in the current position at this health center ___________
16. Is oxygen therapy service provided in the facility?­­­_______
    1. Yes
    2. No

**Questions for assessment of knowledge of HCW**

1. Did you get basic trainings/orientation on oxygen therapy?
   1. Yes
   2. No
2. Have you ever used pulse oximetry to measure oxygen saturation?
   1. Yes
   2. No
3. Which one of the following **is not** a cause of hypoxemia in neonates and children?
   1. Birth asphyxia
   2. Pneumonia
   3. Sepsis
   4. Meningitis
   5. None
4. Clinical signs are not reliable predictors of hypoxemia.­­­­
   1. Yes
   2. No
   3. I don’t know
5. Which one of the following **is not** a sign of hypoxemia in neonates and children?
   1. Inability to drink
   2. fast breathing
   3. Severe lower chest in-drawing
   4. Central cyanosis
   5. None
6. Hypothermia and poor peripheral perfusion will affect the saturation measurement on pulse oximetry.
   1. Yes
   2. No
   3. I don’t know
7. Normal oxygen saturation ranges from 80-98%.
   1. Yes
   2. No
   3. I don’t know
8. When using a pulse oximeter, what cut-off point of oxygen saturation (SPO2) do you use to initiate oxygen therapy?
   1. <70
   2. <80
   3. <90
   4. <98
   5. I don’t know
9. For the usual low-flow rate oxygen therapy such as given via nasal prongs, what standard flow rates do you administer for infants?
   1. O.5L/m – 1L/m
   2. 1L/m – 2L/m
   3. 1L/m – 4L/m
   4. All.
   5. I don’t know
10. Which statement is **NOT true** on administration of oxygen using nasal prongs or catheter in children?
    1. Suitable and better tolerated by patients
    2. Oxygen administration via nasal prongs range from 1-4 litter/minute.
    3. Indicated for use in patients with severe respiratory distress
    4. All
