# Supplementary material for: Decentralizing oxygen availability and use at primary care level for children under-five with severe pneumonia, at 12 Health Centers in Ethiopia: a pre-post non-experimental study
Source: BMC Health Serv Res. 2022 May 19;22:676. doi: 10.1186/s12913-022-08003-4 (PMC9121544; doi:10.1186/s12913-022-08003-4)
Supplement: Supplementary file 3 — Additional file 3. HCW Skill. [file 12913_2022_8003_MOESM3_ESM.docx]

**Section I. 3 Observation questions to assess skills of HCP**

Ask three healthcare providers from under five OPD, labor and delivery and the head of health centre to demonstrate pox and oxygen application on volunteer (another healthcare provider or doll) ***if he/she has ever applied pox and oxygen to patients previously*.**

1. Observe when pox is applied to the volunteer
   - Does he/she able to properly place the POx prob on the finger?
     1. Yes
     2. No
   - Does he/she wait until the wave is regular?
     1. Yes
     2. No
   - Does he/she read the saturation and pulse properly?
     1. Yes
     2. No
   - Does he/she interpreted the reading appropriately?
     1. Yes
     2. No
2. Observe when oxygen is applied to the volunteer
   - Does he/she able to properly place the nasal prong?
     1. Yes
     2. No
   - Does he/she able to set appropriate flow rates for nasal prongs?
     1. Yes
     2. No
   - Does he/she able to do basic preventive maintenances and equipment care?

(cleaning the body, cleaning and replacing the filter, disinfecting the prongs)

- - 1. Yes
    2. No
