# Supplementary material for: Decentralizing oxygen availability and use at primary care level for children under-five with severe pneumonia, at 12 Health Centers in Ethiopia: a pre-post non-experimental study
Source: BMC Health Serv Res. 2022 May 19;22:676. doi: 10.1186/s12913-022-08003-4 (PMC9121544; doi:10.1186/s12913-022-08003-4)
Supplement: Supplementary file 4 — Additional file 4. Medical equipment mgt. [file 12913_2022_8003_MOESM4_ESM.docx]

**Section I. 4 : Medical equipment management**

1. Does the facility have one of the oxygen supply devices (cylinder, concentrator or POx)?
   1. Yes
   2. No
2. If Yes for the above question
   1. Number of cylinders______________
   2. Number of concentrators _____________
   3. Number of POx ­­­­______
3. If yes for ‘’Q1” total number of functional Cylinder in the health center__________________
4. If cylinder is the source of oxygen supply, cost for refiling cylinders per month (including transport)?_______in Birr.
5. If yes for “Q1” total number of functional oxygen concentrator in the health center_________________
6. If you are using oxygen concentrator, do you have reliable alternative power source in case of the direct power interruption?
   1. Yes
   2. No
   3. If yes, for the above question
   4. Generator
   5. Solar sources
   6. Other, please specify ___________________
7. Total number of functional Pulse oximeter in the HC__________________
8. Number of functional Pulse Oximeter in the under 5 OPD___________
9. Does the facility has a position for medical equipment maintenance personnel? _________________
10. If yes, for Q9, do you have a biomedical technician or engineer who is responsible to maintain medical equipment? _________________________
11. If yes for Q10, is he/she trained to maintain oxygen devices ?_______________________
12. If no for Q10, where do you get the maintenance support? ____________________
13. On average, how long does it takes for them to respond to your maintenance support request in days? _________________
14. Do they bring spare parts with them? _________________
15. Does the facility has a budget for procurements of medical equipment? _________________
16. If no, where do you get medical equipment? _______________________
17. Does the facility has a budget for procurements of spare parts? ____________________
18. If no, where do you get spare parts? ___________________________
