# Supplementary material for: Decentralizing oxygen availability and use at primary care level for children under-five with severe pneumonia, at 12 Health Centers in Ethiopia: a pre-post non-experimental study
Source: BMC Health Serv Res. 2022 May 19;22:676. doi: 10.1186/s12913-022-08003-4 (PMC9121544; doi:10.1186/s12913-022-08003-4)
Supplement: Supplementary file 5 — Additional file 5. Medical record review Tool. [file 12913_2022_8003_MOESM5_ESM.docx]

**Section I. 5: Medical record review**

**Part I: Identification**

| ***INSTRUCTIONS****: Complete the sections for sampled charts using the defined sampling procedure and inclusion and exclusion criteria. All charts* ***from Ginbot 1 to Hamle 30, 2011 E.C*** *with clearly stated diagnosis of* ***Birth asphyxia, Very low birth Wight/Very preterm, Very Severe Disease and*** ***PNEUMONIA (ANY TYPE)*** *should be reviewed.* | | | |
| --- | --- | --- | --- |
| **NO.** | **QUESTION** | **RESPONSE CODE** | **SKIP** |
| PXID1 | Date of visit  Ethiopian Calendar (DD/MM/YY) | ____ / ____ / ____ |  |
| PXID2 | Region | Amhara 1  Oromia 2  SNNPR 3  Tigray 4 |  |
| PXID3 | Health Center Name |  |  |
| PXID4 | Data collector’s name |  |  |

**Part II: Patient DATA**

| **NO.** | **QUESTION** | **RESPONSE CODE** | **SKIP** |
| --- | --- | --- | --- |
| PX1 | Record the MRN number  9 = Information missing | MRN number ___ ___ ___ ___ ___ ___ |  |
| PX2 | Record the date of patient visit to HC in Ethiopian calendar (DD/MM/YY) [  *9 = Information missing* | ____ / ____ / ____ |  |
| PX3 | Record the date of discharge  Ethiopian calendar (DD/MM/YY)  *9 = Information missing* | ____ / ____ / ____ |  |
| PX4 | Record the discharge status | Discharged (no mention of status) 1  Discharged cured 2  Improved 3  No change 4  Worse 5  Discharged against medical advice 6  Referred 7  Died 8  Other (specify) 9  Information missing 99 |  |
| PX5 | Record the age of the child | 0-11 months 1  1 year (12-23 months) 2  2 years (24-35 months) 3  3 years (36-47 months) 4  4 years (48-59 months) 5  Information missing 9 |  |
| PX6 | Record the age of the child in months  0 = child is less than 1 month old  99 = Information missing | Age in months/weeks ___ ___ |  |
| PX7 | What is the sex of the child? | Male 1  Female 2  Information missing 9 |  |
| PX8 | Record the child’s weight in kilograms at diagnosis  99 = Information missing | Weight in kg ___ ___. ___ |  |
| PPX9 | Record the child’s length or height in centimetres at diagnosis  *9 = Information missing* | Length/height in cm ___ ___ ___ |  |
| PX10 | Record the child’s body temperature at diagnosis in Celsius  Special codes:    9 = Information missing | Temperature in Celsius ___ ___ ___ |  |
| PX11 | Record the child respiratory rate at diagnosis  Special codes:    999 = Information missing | Respiratory rate ___ ___ ___ |  |
| PX12 | Record all of the signs and symptoms documented by the clinical staff | Cough 1  Tachypnea/Fast breathing 2  Shortness of breath 3  Vomiting 4  Not able to drink 5  Seizure/Convulsions 6  Fever 7  Nasal flaring 8  Grunting 9  Cyanosis 10  Chest in-drawing/retractions 11  Lethargic/unconscious 12  Stridor 13  Diarrhoea 14  Severe malnutrition (SAM) 15  Other (*specify*) 16  None (no symptoms recorded) 17 |  |
| PX13 | Is oxygen saturation (SpO2), recorded for the patient at diagnosis (triage and/or at initial assessment)? | Yes 1  No 2 | 2🡪PX15 |
| PX14 | What was the oxygen saturation (SpO2) reading recorded at diagnosis (take at diagnosis if both triage and diagnosis values exist)? | ________________________________ |  |
| PX14.1 | Was the SpO2 < 90% (hypoxemic)?  ** < 94% for severe sepsis, severe anaemia, shock and heart failure* | Yes 1  No 2 |  |
| PX15 | Was oxygen prescribed for the child? | Yes 1  No 2 | 2🡪PX19 |
| PX16 | What was the flow rate prescribed?  *Special codes:*  *99 = information missing* | # litres per minute ___ ___ |  |
| PX17 | What was the target saturation stated?  *Special codes:*  *99 = information missing* | Target SPO2 ___ ___ ___ % |  |
| PX18 | What was the monitoring frequency advised?  *Special codes:*  *99 = information missing* | SPO2 monitoring frequency ______________ |  |
| PX19 | Is there any recorded oxygen saturation (SpO2) at any point after diagnosis?   - *This is excluding the record at diagnosis* | Yes 1  No 2 |  |
| PX20 | What was the lowest oxygen saturation (SpO2) reading recorded? | Lowest SPO2 reading ___ ___ ___ . ___% |  |
| PX21 | What was the highest SpO2 reading recorded? | Highest SPO2 reading ___ ___ ___. ___% |  |
| PX22 | Did the child have SPO2 < 90 during any measurement after diagnosis? | Yes 1  No 2 |  |
| PX23 | Did the child receive oxygen? | Yes 1  No 2 | 2🡪PX27 |
| PX24 | In total, how many records of SpO2 are found during the period the child was on oxygen ?  *This includes the record at diagnosis* | No. (SpO2) records ___ ___ |  |
| PX25 | Is oxygen saturation (SpO2) recorded for the patient at discontinuation of oxygen therapy? | Yes 1  No 2 |  |
| PX26 | What was the saturation (SpO2) at the time of discontinuation of oxygen therapy? | __________________________________ |  |
| PX27 | Were any antibiotics prescribed for the child? | Yes 1  No 2 |  |
| PX28 | Write any comments you have | ______________________________________________________________________________________________________________________________________________________________________________________________________________________ |  |
