# Supplementary material for: Decentralizing oxygen availability and use at primary care level for children under-five with severe pneumonia, at 12 Health Centers in Ethiopia: a pre-post non-experimental study
Source: BMC Health Serv Res. 2022 May 19;22:676. doi: 10.1186/s12913-022-08003-4 (PMC9121544; doi:10.1186/s12913-022-08003-4)
Supplement: Supplementary file 6 — Additional file 6. Checklist for Chart Review. [file 12913_2022_8003_MOESM6_ESM.docx]

Hospital Name__________________ Date of visit ___________________

Review 10 randomly selected medical records of under 5 patients with severe pneumonia seen in the past 6 months:

|  | Criteria | Write 1 if Yes, write 2 if No or write 3 if not applicable (the stated service is not available) | | | | | | | | | |
| --- | --- | --- | --- | --- | --- | --- | --- | --- | --- | --- | --- |
|  |  | **MR-1** | **MR-2** | **MR-3** | **MR-4** | **MR-5** | **MR-6** | **MR-7** | **MR-8** | **MR-9** | **MR-10** |
| 1 | Did the child get a POx assessment at triage? |  |  |  |  |  |  |  |  |  |  |
| 2 | Did the child get a POx assessment on diagnosis? |  |  |  |  |  |  |  |  |  |  |
| 3 | Did the child have hypoxemia (SPO2 < 90) at diagnosis? |  |  |  |  |  |  |  |  |  |  |
| 4 | Was the child prescribed oxygen as part of the initial clinician’s  order at diagnosis? |  |  |  |  |  |  |  |  |  |  |
| 5 | Did the child get a POx assessment at any point after diagnosis? |  |  |  |  |  |  |  |  |  |  |
| 6 | Did the child have hypoxemia (SPO2 < 90) at any point after diagnosis? |  |  |  |  |  |  |  |  |  |  |
| 7 | Was the child prescribed oxygen at any time (including at diagnosis)  during the stay (emergency and/or IPD)? |  |  |  |  |  |  |  |  |  |  |
| 8 | If yes to Q7, does the prescription state the mode of delivery of oxygen?  (Nasal prong, Catheter or Facemask) |  |  |  |  |  |  |  |  |  |  |
| 9 | If yes to Q7, does the prescription state flow rate? |  |  |  |  |  |  |  |  |  |  |
| 10 | If yes to Q7, does the prescription mention target SPO2? |  |  |  |  |  |  |  |  |  |  |
| 11 | If yes to Q7, did the prescriber mention frequency of monitoring SPO2? |  |  |  |  |  |  |  |  |  |  |
| 12 | If Yes to Q7, did the child actually receive oxygen? |  |  |  |  |  |  |  |  |  |  |
| 13 | Was the oxygen therapy stopped after at least two records of SPO2 > 90%? |  |  |  |  |  |  |  |  |  |  |
